# Partial Characterization of Venom from the Colombian Spider *Phoneutria Boliviensis* (Aranae:Ctenidae)

**DOI:** 10.3390/toxins7082872

**Published:** 2015-07-31

**Authors:** Sebastian Estrada-Gomez, Leidy Johana Vargas Muñoz, Paula Lanchero, Cesar Segura Latorre

**Affiliations:** 1 Facultad de Ciencias Farmacéuticas y Alimentarias, Universidad de Antioquia UdeA, Medellín 050010, Colombia; 2 Programa de Ofidismo/Escorpionismo, Facultad de Ciencias Farmacéuticas y Alimentarias, Universidad de Antioquia UdeA, Medellín 050010, Colombia; E-Mail: paula.lanchero@gmail.com; 3 Facultad de Medicina, Universidad Cooperativa de Colombia, Medellín 050010, Colombia; E-Mail: leidy.vargasmu@campusucc.edu.co; 4 Unidad de espectrometría de masas, Sede de Investigación Universitaria, Universidad de Antioquia UdeA, Medellín 050010, Colombia; E-Mail: cesars79@gmail.com

**Keywords:** *Phoneutria boliviensis*, ctenidae, RP-HPLC, insecticidal activity, mass analysis, proteomic analysis, phospholipases

## Abstract

We report on the first studies on the characterization of venom from *Phoneutria boliviensis* (Aranae:Ctenidae) (F. O. Pickard-Cambridge, 1897), done with Colombian species. After the electrostimulation extraction process, the venom showed physicochemical properties corresponding to a colorless and water-soluble liquid with a density of 0.86 mg/mL and 87% aqueous content. *P. boliviensis* venom and RP-HPLC fractions showed hemolytic activity and hydrolyzed the synthetic substrate 4-nitro-3-octanoyloxy-benzoic acid, indicating the presence of phospholipases A_2_ enzymes. The electrophoretic profile showed an important protein content with molecular masses below 14 kDa, and differences between male and female protein content were also revealed. The RP-HPLC venom profile exposes differences between males and female content consistent with the electrophoretic profile. Five fractions collected from the RP-HPLC displayed significant larvicidal activity. Mass analysis indicates the presence of peptides ranging from 1047.71 to 3278.07 Da. Two peptides, Ctenitoxin-Pb48 and Ctenitoxin-Pb53, were partially identified using HPLC-*n*ESI-MS/MS, which showed a high homology with other Ctenitoxins (family Tx3) from *Phoneutria nigriventer*, *Phoneutria keyserlingi* and *Phoneutria reidyi* affecting voltage-gated calcium receptors (Cav 1, 2.1, 2.2 and 2.3) and NMDA-glutamate receptors.

## 1. Introduction

Worldwide, the genus *Phoneutria* is made up of five different species mainly known for their potent neurotoxic venom and aggressive behavior [1,2,3,4]. In Colombia, *P. reidyi*, *P. fera* and *P. boliviensis* have been reported. *Phoneutria boliviensis* (F. O. Pickard-Cambridge, 1897) is a widely distributed species on the American continent and can be found in South America, spread amongst Bolivia, Paraguay, Peru, Ecuador, Brazil, and Colombia [4]. In Colombia, this species has been reported in the Antioquia and Magdalena provinces [1,4,5] and nowadays its distribution has expanded to Boyacá, Caldas, Cauca, Cundinamarca, Quindío, Risaralda, Santander, and Valle del Cauca [5].

Venom production in spiders have played a decisive role in the long-term evolution of this group of living organisms, since it has provided them with a highly toxic arsenal not only to kill or paralyse their prey but also to act as a repellent against aggressors [6]. Spider venoms are heterogeneous mixtures of biological active compounds; up to one thousand different components have been reported in just one species’ venom [7]. These components include salts, nucleotides, peptides, proteins, and enzymes which affect both vertebrates and invertebrates [6,8,9,10,11]. These heterogeneous mixtures are considered a rich source of low molecular mass compounds displaying antimicrobial activities [12], with the most abundant reports of neurotoxic peptides with high contents of cysteine (Cys) residues, affecting a great variety of presynaptic ion channels [13]. These peptide-like components can be divided into three groups: low molecular mass components (<1 kDa–acylpolyamines and linear cytolytic peptides), medium molecular mass components (<10 kDa), and high molecular mass components (>10 kDa) [14] enhancing neurotoxic or cytotoxic activity [6,15,16].

To date, around 400 peptides and proteins, ranging from 1.2 to 27 kDa, have been isolated from the genus *Phoneutria* in a pure state and, out of these, nearly 100 have had their complete or partial amino acid sequences determined [17], although most studies have been focused on the species *P. nigriventer* and *P. reidyi*. The purified reported peptides belong to the *P. nigriventer*, *P. reidyi and P. keyserlingi* venoms and there are no available reports from the *P. boliviensis* venom. Some of these peptides act as neurotoxins on the glutamate receptors of the central nervous system and/or in neural ion channels [18,19]. From *P. nigriventer*, five toxic fractions have been isolated targeting both mammals and insect ionic channels. Fractions 1, 2 and 3 were identified to contain different proteases, while fraction 4 showed four main peaks PhTx1, PhTx2, PhTx3 and PhTx4, containing polypeptides and enhancing different toxicological effects [19,20]. PhTx2 was found to inhibit Na^+^ channel inactivation and evoke acetylcholine release [20,21,22], and activation of the Na^+^ channel in the rat phrenic nerve-diaphragm, through four cysteine-rich variants of PhTx2 named Tx2-1, Tx2-5, Tx2-6, Tx2-9 of 5838, 5116, 5291 and 3742 Da, respectively [23,24]. PhTx3 is a mixture of at least six isotoxins been Tx3-3 a potent inhibitor of ω-agatoxin IVA sensitive Ca^2+^ channels [25] and Tx3-1 a selective inhibitor of the A-type K^+^ channels [26]. All recent studies reveal that *Phoneutria* genus venoms are very similar concerning peptide content, although some specific qualitative and quantitative differences can be detected [17].

At present, spiders have become the primary focus of various research efforts due to the wide range of species described so far, their broad distribution, and the complex and variable composition of their venoms. Most of all, the *P. boliviensis* has become an interesting topic of study given the absence of information reported for this species and its venom composition; thus, in this work the chromatographic, electrophoretic and biochemical profile of *P. boliviensis* venom is reported.

## 2. Results

### 2.1. Venom Physicochemical Properties

After the female and male milking process, a colorless and low-viscosity venom was obtained with a density of 0.86 mg/mL. The overall general wet weight media obtained for the pooled venom was 16.7 ± 5.23 mg, while the dry weight was 2.8 ± 1.11 mg. After the lyophilization process, a white solid 100% soluble in aqueous solvents was obtained. The overall venom aqueous content percentage (v.aq.p.) was 87% ± 0.01%. Body size showed a sexual dimorphism between males and females that correlated with the amount of venom yielded by each one, with higher amounts of venom observed from females. Male and female comparison parameters are shown in Table 1.

**Table 1:** *Phoneutria boliviensis* male and female venom physicochemical properties comparison. **^‡^** indicates values with *p <* 0.05; * indicates values with *p >* 0.05. CF: Cephalotorax measures. *L*: length and *W*: width.

	*Phoneutria boliviensis*	
	Male	Female
**Wet weight ^‡^**	9.7 ± 4.36 mg	20.9 ± 7.68 mg
**Dry weigh ^‡^**	1.4 ± 0.92 mg	3.7 ± 1.65 mg
Aqueous content *	89% ± 0.03%	85% ± 0.02%
**CF Measures ^‡^**	**L:** 12.03 ± 0.69 mm **W:** 9.6 ± 0.29 mm	**L:** 14.36 ± 0.39 mm **W:** 10.65 ± 0.21 mm

### 2.2. Enzymatic Properties

**Figure 1:**
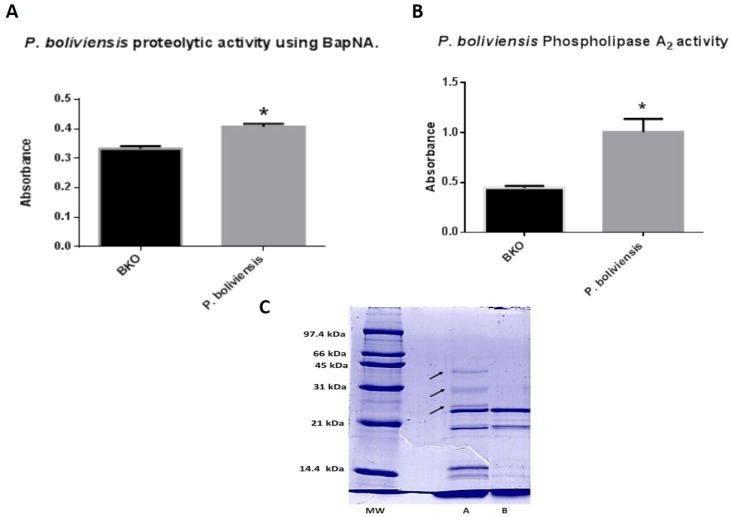
(**A**) Phospholipase A_2_ activity using the substrate 4-nitro-3-octanoyloxy-benzoic acid with 100 µg of *P. boliviensis*; (**B**) Proteolytic activity using the substrate *N*-benzoyl-DL-arginine-p-nitroanilide (BapNA) with 100 µg of *P. boliviensis* venom. Asterisks indicates significant differences with *p <* 0.05 with respect to blanks (BKO); (**C**) *P. boliviensis* crude venom (reduced) SDS-PAGE profile in a 12% gel followed by Coomassie blue staining. Venoms were loaded at a concentration of 0.5 mg/mL. F: Female, M: Male, MW: Molecular weight markers. Arrows indicate absence of high molecular weight components in males.

Venom showed indirect hemolytic activity with and without calcium, and the minimum hemolytic dose (MHeD) was set at 202.5 µg. This activity was confirmed to be dependent on enzymatic active phospholipases A_2_ (PLA_2_) since the complete venom hydrolyzed the specific PLA_2_ substrate, showing statistically significant differences with respect to blank with a *p <* 0.05 (Figure 1A). Moreover, two RP-HPLC fractions eluting at 52 and 57.5 min (33% and 37% of acetonitrile, respectively), showed the same specific PLA_2_ activity. *P. boliviensis* venom, showed proteolytic activity, degrading both azocasein and *N*-benzoyl-DL-arginine-p-nitroanilide (BapNA) substrates (Figure 1B), and only staitistically significant differences were found with respect to blank in the BapNA assay (*p <* 0.05). BapNA activity was found only at a concentration of 100 µg. No signif8cant coagulant activity was observed with this venom (data not shown).

### 2.3. SDS-PAGE

The *Phoneutria boliviensis* venom SDS-PAGE profile (Figure 1C) reveals similarities in the existence of low and medium molecular mass proteins in males and females; it reveals the presence of bands in the range from 14 to 45 kDa. A significant concentration of bands close to 14 kDa is observed in both venoms but they appear to be lighter in male venom. Three bands above 25 and below 45 kDa can be observed in female venom but they appear to be absent from male venom.

### 2.4. Reverse-Phase Chromatography

**Figure 2:**
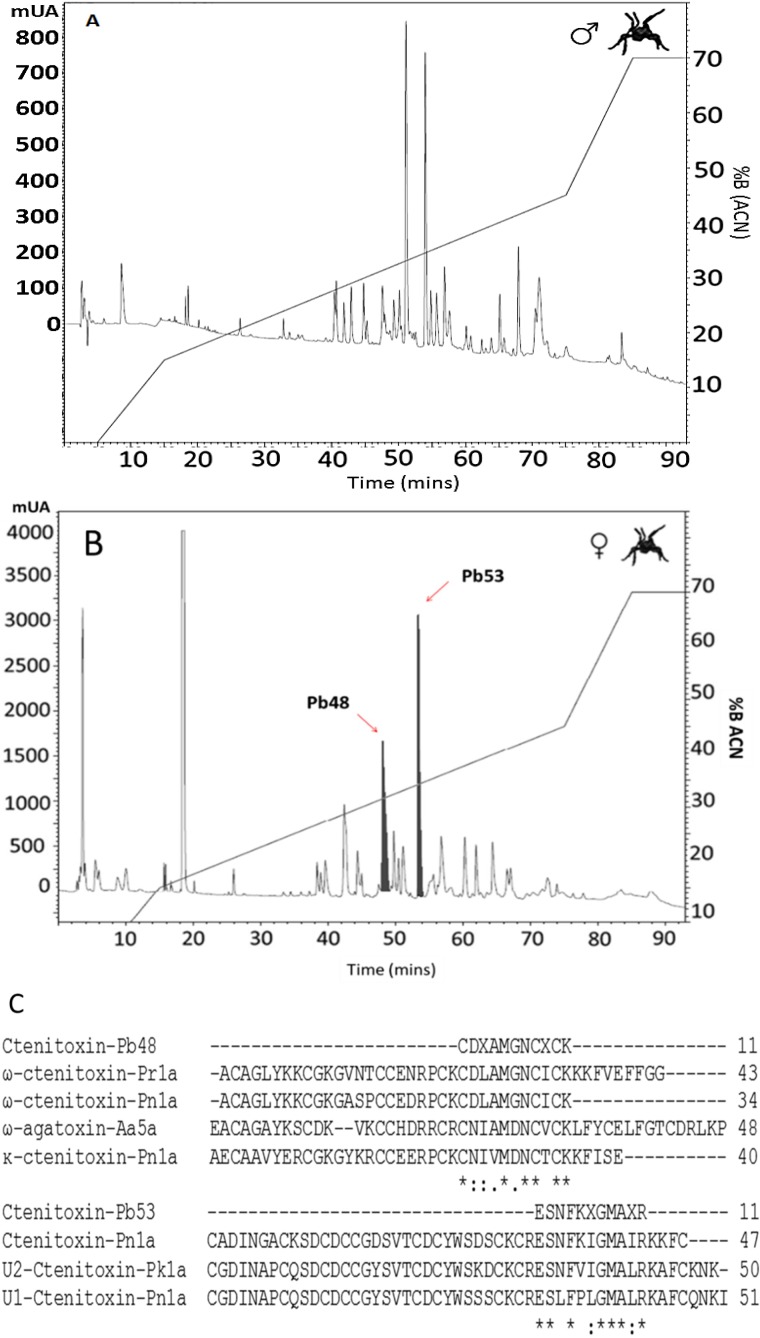
*P. boliviensis* venom (1 mg) RP-HPLC chromatographic profile using a C18 column (250 × 4.6 mm). A 0%–70% of acetonitrile (99% in TFA 0.1%) elution gradient was used. The run was monitored at 215 nm. (**A**) Male and (**B**) female; (**C**) alignment of Ctenitoxin-Pb48 and Ctenitoxin-Pb53 peptides with other sequences. Internal peptides obtained after tryptic digestion of *P. boliviensis* Ctenitoxins and tandem mass spectrometry analysis, as described in materials and methods, are aligned with the following Ctenidae peptides: ω-ctenitoxin-Pn1a (UniProtKB:O76201), κ-ctenitoxin-Pn1a (UniProtKB:O76200), U1-Ctenitoxin-Pn1a (UniProtKB:P61229) and Γ-Ctenitoxin-Pn1a (UniProtKB:P59367) from *P. nigriventer*; U2-Ctenitoxin-Pk1a (UniProtKB:P83905) from *P. keyserlingi*; ω-ctenitoxin-Pr1a (UniProtKB:P83911) from *P. reidyi*. From the Agenelidae family, a sequential homology was found with ω-agatoxin-Aa5a (US Patent number 5599559A).

The reverse phase high-performance liquid chromatography (RP-HPLC) profile obtained from *Phoneutria boliviensis* male venom showed 27 well-defined peaks (Figure 2A), while the female venom chromatographic profile showed 29 well-defined peaks (Figure 2B). From the female’s run, two collected peaks were identified through a HPLC-*n*ESI-MS/MS system.

### 2.5. LC–MS Analysis

Table 2 lists the observed masses present in selected RP-HPLC fractions (see also Figure 2B). A total of 25 molecular species were observed in the selected fractions with isotopic masses ranging from 1047.71 to 3278.07 Da.

**Table 2:** Molecular masses of each collected peak from female’s RP-HPLC venom in Figure 2B and determined by Orbitrap indicated by [*M* + *H*]+. Values show isotopic and average molecular masses in daltons (Da) and the charge (Z). rT: Retention time.

rT (min)	Isotopic Mass (Da)	Average Mass (Da)	*Z*	rT (min)	Isotopic Mass (Da)	Average Mass (Da)	*Z*
3.6	1271.90	1273.92	+2	39.5	1376.97	1378.98	+2
	1529.09	1531.09	+2		1573.12	1574.12	+3
	1599.14	1601.12	+3		1617.15	1618.15	+3
	1617.15	1619.15	+3		2937.37	2939.38	+5
	2939.38	2941.40	+5		2994.44	2997.44	+5
	2993.44	2996.47	+5		3180.48	3182.49	+5
33.3	2511.59	2513.60	+4	42.3	1114.82	1115.83	+2
	2939.38	2941.40	+5		1118.75	1119.75	+2
	3180.48	3183.50	+5		1467.04	1469.04	+2
34.4	1047.71	1048.75	+2		1493.04	1496.07	+2
	1070.80	1071.80	+2	60.2	1467.03	1469.04	+2
	1398.81	1400.91	+2		3180.50	3183.50	+5
	1511.06	1513.07	+2	61.9	1510.04	1511.06	+2
35.9	1557.12	1558.13	+3		1617.15	1619.15	+3
	1644.17	1646.18	+3		3180.50	3183.50	+5
	2467.56	2469.57	+4				
	2511.58	2513.62	+4				
	3278.07	3279.06	+5				

### 2.6. Peptide Identification

Pb48 and Pb53 collected fractions were identified using the HPLC-*n*ESI LC/MS system. The identified peptides were named Ctenitoxin-Pb48 and Ctenitoxin-Pb53 according to the nomenclature recommended by King *et al.* [27]. The BLAST search of the identified peptides on this work led to the determination of homology among them and some neurotoxins identified in other Brazilian species of the *Phoneutria* genus such as the ω-ctenitoxin-Pn1a (UniProtKB:O76201), κ-ctenitoxin-Pn1a (UniProtKB:O76200), U1-Ctenitoxin-Pn1a (UniProtKB:P61229) and Γ-Ctenitoxin-Pn1a (UniProtKB:P59367) from *P. nigriventer*; U2-Ctenitoxin-Pk1a UniProtKB:P83905) from *P. keyserlingi*; and ω-ctenitoxin-Pr1a (UniProtKB:P83911) from *P. reidyi*. Sequential homology was also found with the neurotoxin ω-agatoxin-Aa5a (Patent number US 5599559A) identified in the *Agelenopsis aperta* venom, which belongs to the Agelenidae family (Figure 2B,C, and Table 3). Table 3 shows the homology percentage obtained for each toxin.

**Table 3:** Protein peptide/summary of the identified peptides. MM: calculated molecular mass. Aminoacid homology (AA % ID) is from clustalW. All database numbers from UniProtKB.

Fraction	Monoisotopic mass MH+	*Z*	MS/MS derived sequence	Data base ID. All UniProtKB	AA % ID	Species	Matched peptide
Ctenitoxin-Pb48	1341.542	+2	CDXAMGNCXCK	P83911	100	*Phoneutria reidy*	ω-ctenitoxin-Pr1a (Tx3-7)
				O76201	100	*Phonuetria nigriventer*	ω-ctenitoxin-Pn1a (Tx3-2)
				O76200	54	*Phonuetria nigriventer*	κ-ctenitoxin-Pn1a (Tx3-1)
Ctenitoxin-Pb53	1265.667	+3	ESNFKXGMAXR	P83905	100	*Phoneutria keyserlingi*	U2-Ctenitoxin-Pk1a (U2-CNTX-Pk1a)
				P61229	81	*Phonuetria nigriventer*	U1-Ctenitoxin-Pn1a (U2-CNTX-Pn1a)
				P59367	63	*Phonuetria nigriventer*	Ctenitoxin-Pn1a (Tx4(5-5))

### 2.7. Larvicidal Activity

From the RP-HPLC fractions evaluated on *Aedes aegypti* larvae, only the peaks eluting at 19.6, 22.4, 64.6, 66.9 and 68.1 min showed partial larvicidal activity after 24 h. Most effective results were obtained with fractions eluting after 60 min, were 80% to 100% of larvae showed high degree of ataxia and posterior death (data not shown).

## 3. Discussion

This is the first report of partial characterization of venom from the Ctenidae spider *P. boliviensis*. There is a growing interest in venom from different arachnids since they represent a wide pharmacological library of biomolecules, especially in the *Phoneutria* genus due to the toxicological effects of this venom in mammals. In spite of the interest around these biomolecules, access to this biological resource has been problematic, mainly because of the aggressiveness of some species during venom extraction, which can only be obtained by direct milking, electrical stimulation, or by direct extraction from the venom gland. The venom extraction process followed in this work avoided its contamination with gastric fluids or hemolymph, since the only body parts in contact with the syringe were the fang tips. The amount of venom yielded after each extraction process showed a significant difference among males and females, as seen in other species such as *Pamphobeteus* aff *nigricolor* [16]. In all cases, females yielded higher amounts of venom (wet or dry). This is probably due to sexual size dimorphism observed in these species, due to which the females usually have bigger bodies than males [2,28]. Aqueous content percentage was significantly higher in males than females, which is in concordance with the results observed in the electrophoresis and chromatography, where females showed an apparently higher amount of proteins and higher numbers of peaks, respectively.

The enhanced indirect hemolytic activity showed a MHeD of 202.5 µg. This activity is confirmed as dependent on the presence of PLA_2_ since the complete venom and two fractions eluting at 51.8 and 57.5 min hydrolyzed the specific substrate 4-nitro-3-octanoyloxy-benzoic acid. The electrophoretic and chromatographic profile confirms the presence of compounds with similar molecular masses of these phospholipases, bands around 14 kDa, and different peaks eluted in the arachnids’ phospholipase region, between 25% and 30% acetonitrile, as described in different arachnids [16,29,30,31]. More specific assays should be performed to determine enzymatic parameters and characterize these enzymes. The proteolytic activity detected indicates, preliminarily, the presence of serine proteases, since the complete venom hydrolyzed the specific substrate BapNA. This kind of proteins in arachnids are responsible for fibronectinolytic and fibrinogenolytic activities, with molecular masses ranging from 85 and 95 kDa, as reported in other arachnids [32]. Specific assays should be performed to determine enzymatic parameters, and structural identification and MS/MS analysis including peptide identification for this kind of enzyme compound must also be performed.

The electrophoretic profile shows a wide distribution of masses and a significant difference between male and female venom composition. Three bands above 25 kDa and below 45 kDa can be observed in female venom but they appear to be absent in male venom. The same happens with bands close to 14 kDa, which are observed in both venoms but appear to be lighter in male venom. Similar intersexual variations have been described before in other *Phoneutria* genera between males and females as in *Pamphobeteus nigricolor*, along with other species [17]. In the venom from *P. boliviensis*, three main groups can be observed: one group corresponding to compounds below 14 kDa, a second one corresponding to compounds with a molecular mass close to 21 kDa (between 21 and 31 kDa) and a third group corresponding to compounds with molecular mass over 31 kDa. A similar “distribution” of masses was observed in the venom form *P. nigricolor*, where compounds up to 45 kDa were detected in addition to a significant concentration of compounds below 14 kDa [17]. It is well known that the majority of the compounds are in the size range of 3–12 kDa with a predominance of cationic peptides [17,33]. Masses over 21 kDa could belong to enzymes involved in toxin production as reported in other spider genera [34], or as explained above, they could belong to certain proteases.

The chromatographic profile allowed the detection of significant differences between males and females. The main difference is observed in compounds eluting between 31% and 35% of acetonitrile, where male venom shows a higher concentration of compounds compared to female venom. Similar differences are observed when comparing the venom form *P. nigriventer*, *P. reidyi* and *P. keyserlingi* simultaneously, founding an evident intraspecific variability in the chromatographic profile region between 32% and 37% of acetonitrile [17]. Other differences are observed in components eluting between 8% and 20% of acetonitrile, where predominant components from female venoms elute in this region (peak eluting at 19 min), while in males, predominant compounds elute above 34% of acetonitrile (peaks eluting at 52 and 54 min). The female’s venom profile shows a predominant peak eluting below 5 min, close to the solvent front, which could correspond to high-polarity peptides, and polarity is weakly retained in the column mentioned above. This peak showed molecular masses as low as 1271.91 Da, consistent with other cationic and high-polarity peptides reported from *Phoneutria nigriventer* and other arachnids, which may correspond to the amphipathic α-helical compounds or cytolytic peptides [12,33,35].

High-resolution mass analysis indicates the presence of about 25 protein components in the selected fractions, with a median of 4 compounds per fraction, and masses ranging from 1047.71 to 3278.07 Da. The masses reported for *P. boliviensis* venom may correspond to the mentioned cationic peptides, or peptides affecting different ionic channels, as have been reported in other *Phoneutria* genera [17,33,36]. As seen below, these data are confirmed with de-MS/MS analysis and the internal sequence peptides found, which show homology with neurotoxic peptides from the Tx3 family, found in other Phoneutrias [17,18,37]. Pimenta *et al.* reported a mass fingerprint from the venom of *P. nigriventer* including identified peptides with molecular masses ranging from 303.310 to 7543.180, with masses very similar to those described in this report for *P. boliviensis*, as seen in Table 2 [33].

Alignment of the internals peptide amino acid sequences using the CC motif from the selected RP-HPLC fractions, Ctenitoxin-Pb48 and Ctenitoxin-Pb53, showed homology with other neurotoxins from different Brazilian species of the *Phoneutria* genus such as *P. nigriventer*, *P. keyserlingi* and *P. reidyi*. Ctenitoxin-Pb48 showed high homology with some members of the Tx3 family. This family had been reported to induce progressive flaccid paralysis in mice legs followed by death, some of them affecting Ca^2+^ channels (L, N and P/Q type) and K^+^ channels [17,18]. They have a molecular mass range from 3500 to 8500 Da (3549.0 Da for Tx3-2, 8449.6 Da for Tx3-4, 5063.6 Da for Tx3-5 and 6017.9 Da for Tx3-6), around 14 cysteine residues (7 disulphide bridges) and structural similarities among their members [18]. Specifically, ctenitoxin-Pb48 showed homology with ω-ctenitoxin-Pr1a (Tx3-7, (UniProtKB:P83911)) from *P. reidyi*, ω-ctenitoxin-Pn1a (Tx3-2, (UniProtKB:O76201)) from *P. nigriventer* and κ-ctenitoxin-Pn1a (Tx3-1, (UniProtKB:O76200)) from *P. nigriventer*. Additionally, Ctenitoxin-Pb48 showed homology with ω-agatoxin-Aa5a, a toxin from *Agelenopsis aperta* (Agelenidae), a specific neurotoxin affecting L- and N-type Ca^2+^ channels. Ctenitoxin-Pb53 showed homology with a Tx4 family member (Tx4- 5-5; Γ-Ctenitoxin-Pn1a (UniProtKB:P59367)). These family members were purified to homogeneity from PhTx4 fraction described in *P. nigriventer* venom, and is characterized by the inhibition of L-Glutamate receptors and their insecticidal activities [19]. Specifically, Tx4 5-5 was shown to inhibit selectively the NMDA-subtype of ionotropic glutamate receptors in rat’s brain [38]. The other homology corresponded to U1-Ctenitoxin-Pn1a (UniProtKB:P61229) from *P. nigriventer* and U2-Ctenitoxin-Pk1a (UniProtKB:P83905) from *P. keyserlingi*. However, the molecular targets of these two toxins are unknown. All this information suggests the presence of toxins affecting different Ca^2+^ channels and probably L-Glutamate receptors.

Peaks eluting at 19.6, 22.4, 64.6, 66.9 and 68.1 min showed partial larvicidal activity on *Aedes aegypti* larvae, after 24 h. This larvicidal activity was characterized by ataxia (and posterior death) observed in the larvae. The induced ataxia and indirect death was due to the low concentration of the fraction collected from the RP-HPLC. Insecticidal activity has been widely reported in other *Phoneutria* genera especially in *P. nigriventer* where the Tx4 family toxins have been identified as responsible for this activity [19] along with other different arachnids such as the scorpion *Centruroides edwardsii* [29]. This activity is most probably mediated through actions in the glutamatergic synapses in the neuromuscular junctions [19].

## 4. Materials and Methods

### 4.1. Spiders

Three males and two females of *Phoneutria boliviensis* from the Urabá region were kept in captivity in the Universidad de Antioquia arachnidarium. The micro-habitat consisted of 35 × 25 × 10 cm plastic boxes provided with water and food *ad libitum* with a polyphagic diet (vertebrates and invertebrates). Median temperature of 26 °C, twelve hours day-light and 12 h of darkness for the photoperiod were proportionate.

### 4.2. Venom Extraction

Due to the animals’ aggressiveness, it was necessary to anesthetize the spiders before the venom extraction and the process proposed by Estrada and co-workers was followed [16] allowing complete recovery of the animal; anesthesia was achieved in a chamber under an isoflurane USP (Baxter, Deerfield, IL, USA) atmosphere for no more than 1.5 min. Once anesthetized, spiders were manually immobilized. Electro-stimulation was applied on each chelicerae electrical stimulus of 35 V applied twice in 5 s intervals using a JRM electro-stimulator (model 06, series 007, Cali, Colombia). Milking processes were carried out every 2 months, 3–4 weeks after feeding. Venom from each individual was collected in syringes and transferred to dry capillars and volume was measured. Subsequently, venom was transferred to a 0.5 mL vial, weighted before and after venom transfer to determine dry and wet weight. After this, venom was lyophilized and stored at –20 °C until used.

### 4.3. Physicochemical Properties

Some venom physicochemical properties were analyzed including color, density, aqueous content and solubility. Color, aqueous content and solubility were determined by visual perception from different evaluators. Density was estimated from the venom wet weight and the venom volume obtained after each milking.

### 4.4. Indirect Hemolytic Activity

This activity was determined in agarose erythrocyte-egg yolk gels, according to Gutiérrez *et al.* [39], using 0.8% agarose dissolved in PBS (0.12 M NaCl, 0.04 M sodium phosphate in distilled water), pH 7.2 and CaCl_2_. An additional plate without CaCl_2_ was performed to verify that the hemolytic activity is due to the phospholipases’ (PLA_2_) presence. The amount of *P. boliviensis* female venom that induced a 20 mm diameter hemolytic halo was defined as minimum hemolytic dose (MHeD). The experiments were run in triplicate.

### 4.5. PLA_2_ Activity

PLA_2_ activity was measured using the assay described by Cho and Kézdy [40] and Holzer and Mackessy [41], modified for 96-well plates. The standard assay mixture contained 200 μL of buffer (10 mMTris-HCl, 10 mM CaCl_2_, 100 mMNaCl, pH 8.0), 20 μL of substrate 4-nitro-3-octanoyloxy-benzoic acid, 20 μL of water and 20 μL of the female venom at a concentration of 5 µg/μL, and the RP-HPLC collected fractions, for a final volume of 260 μL. The mixture was incubated 60 min at 37 °C, and the absorbance was recorded at 405 nm. The experiments were performed in triplicate.

### 4.6. Proteolytic Activity

Azocasein (Sigma-Aldrich, St. Louis, MO, USA) was used as substrate to measure the proteolytic activity, and determine the presence of metalloproteases, according to Wang *et al*, [42] with some modifications. Briefly, 100 to 120 μg of *P. boliviensis* female venom were dissolved in 20 μL of 25 mM Tris (0.15 M NaCl, 5 mM CaCl_2_), pH 7.4, these solutions were incubated with 10 mg/mL of azocasein diluted in the same buffer. After an incubation of 90 min at 37 °C, the reaction was stopped by the addition of 200 μL of trichloroacetic acid. Samples were then centrifuged at 360 *g* for 5 min. Supernatant (100 μL) was mixed with the equal volume of 0.5 M NaOH, and the absorbances were measured (450 nm). Results are expressed as unit of proteolytic activity, which corresponds to the amount of enzyme that induces a change in absorbance of 0.2. The experiments were performed in triplicate.

A second proteolytic activity was performed to determine the presence of serine protease enzymes following the method described by Patiño *et al.* [43]. The enzymatic activity was measured using the synthetic substrate BapNA. The standard assay mixture contained 50 μL of buffer (10 mM Tris-HCl, 10 mM CaCl_2_, 100 mM NaCl, pH 8.0), 200 μL of substrate, 10 μL of water or enzyme in a final volume of 260 μL. After the addition of 100 µg and 50 µg of *P. boliviensis* female venom, the mixture was incubated for up to 40 min at 37 °C, with the absorbance at 415 nm being recorded at 10 min intervals. The experiments were performed in triplicate.

### 4.7. Electrophoretic Profile

According to Laemmli [44] *P. boliviensis* male and females crude venoms were analyzed by sodium dodecyl sulfate polyacrylamide gel electrophoresis or SDS-PAGE, on 12% gels, and stained with Coomassie blue R-250. Molecular weights were estimated using standard markers (Bio-Rad, Hercules, CA, USA).

### 4.8. Chromatographic Profile

One milligram of whole male and female *P. boliviensis* venom was dissolved in 200 μL of solution A (0.1% TFA in water) and centrifuged at 2300 *g*. The supernatant was then applied to a reverse-phase RESTEK C18 column (250 × 4.6 mm), and separated by RP-HPLC on a Shimadzu Prominence chromatograph (Kyoto, Japan). Proteins were eluted by a gradient towards solution B (0.1% TFA in acetonitrile) as follows: 5% B for 5 min, 5%–15% B over 10 min, 15%–45% B over 60 min, and 45%–70% B over 12 min at a flow rate of 1.0 mL/min [45]. The chromatographic run was monitored at 215 nm, fractions were collected, dried using an eppendorf Vacufuge plus vacuum concentrator (Hamburg, Germany) and stored at −20 °C until used.

### 4.9. Peptide Mass Determination by High-Resolution LC-MS

Eight selected fractions (eluting at 3.6–33.3–34.4–35.9–39.5–42.3–60.2, and 61.9 min) corresponding to peaks with the best resolution and representativeness in the chromatogram were collected from female RP-HPLC venom, dried as described above and used to complete mass analysis in a Q Exactive Q-Orbitrap (Thermo Scientific, San Juan, CA, USA) linked to a UHPLC. For the chromatographic step, an analytical C18 Hypersil Gold Aq™ column (Thermo Scientific, San Juan, CA, USA) (2.1 mm internal diameter, 100 mm length and 1.9 µm pore size) was used. Venom components were eluted using gradients of solution B (0.1% formic acid in acetonitrile) as follows: 5% B for 2 min, 5%–90% B over 13 min, 90% B for 2 more min, and 5% B for 5 more min at a flow rate of 450 µL/min.

For the spectrometric assay, ESI source was employed at 3 kV and 320 °C in positive ion mode. Sheath gas flow rate was set at 40 units and auxiliary gas flow rate was set at 10 units. Full scan mode MS spectra (160–2000 m/z) were obtained in the Orbitrap with a resolution *R* = 70,000, without fragmentation. Manual deconvolutions were used to determine the isotopic and average molecular mass composition of the components.

### 4.10. Peptide Identification by LC-MS/MS

Ten micrograms of sample (two well-defined and significant peaks eluting at 48 and 53 min) were reduced with 100 mM DTT, alkylated with 100 mM iodoacetamide and then trypsinized. Digestion was allowed to proceed for 16 h at 30 °C. Protein identification was performed by HPLC-*n*ESI-MS/MS on a C18 nano-column using a 1200 Agilent chromatograph coupled to a 6310 IonTrap mass spectrometer (Agilent). Resulting spectra were analyzed using Spectrum Mill (Agilent Technologies, Santa Clara, CA, USA) and Mascot (MatrixScience, Boston, MA, USA) in the NCBInr protein databases, with carbamidomethylation as fixed modification. Peptide sequences were searched for similarity using BLAST (http://blast.ncbi.nlm.nih.gov/Blast.cgi?PAGE=Proteins) and the Arachnoserver database [13,30] (http://www.arachnoserver.org/mainMenu.html), and aligned with related arachnid peptides using ClustalW [46].

### 4.11. Larvicidal Activity

The method described by Estrada and co-workers [29] was followed with a few modifications. Fifty microliters of *P. boliviensis* reconstituted female RP-HPLC fractions were added to a tube containing 450 μL of saline solution, 0.90%, and 5 *Aedes aegypti* larvae. A 0.90% saline solution was used as a negative control, whereas a solution of piperazine was used as a positive control. The solutions were kept at room temperature 12 h in a light and dark photoperiod. A dead larvae was considered those without movement or ataxic (moribund larvae showing no typical erratic movement of normal larvae). The counting of dead larvae (larvae with no movement) was performed on each tube at 24 h to 48 h. This procedure was performed in duplicate, with approval of the Universidad de Antioquia animal ethical committee.

### 4.12. Statistical Analysis

Results were expressed as mean ± standard error media (S.E.M.) and statistical comparisons were done using an ANOVA with a Bonferroni post-test assuming a significance of *p <* 0.05. All data analysis was done using GraphPad PRISM 5 (GraphPad Software, Inc.; La Jolla, CA, USA).

## 5. Conclusions

In conclusion, *P. boliviensis* venom exhibited similarities to other *Phoneutria* genera venoms such as *P. nigriventer, P. reidyi and P. keyserlingui*, specifically with the neurotoxic Tx3, a statement that allows us to suggest that *P. boliviensis* venom may act by affecting some Ca^2+^ sub-types and glutamate receptors. Furthermore, *P. boliviensis* contain proteins enhancing phospholipase A_2_ activity and proteolytic activity (like metallo and serine proteases). Mass analysis shows similarities with other *Phoneutria* species such as the peptides found in *P. nigriventer* with cationic amphipathic activity.
